# A Multi-Site Assessment of Anesthetic Overdose, Hypothermic Shock, and Electrical Stunning as Methods of Euthanasia for Zebrafish (*Danio rerio*) Embryos and Larvae

**DOI:** 10.3390/biology11040546

**Published:** 2022-04-01

**Authors:** Jean-Philippe Mocho, Florian Lang, Guillaume Valentin, Sébastien Bedu, Robin McKimm, Juan Ramos, Yolanda Saavedra Torres, Sarah E. Wheatley, Joseph Higgins, Mollie E. Millington, Pia Rengtved Lundegaard, Rubén Chamorro Valverde, Vlasta Jenčič, Kristine von Krogh

**Affiliations:** 1 Joint Production System Ltd., Potters Bar EN6 3DD, UK; 2 Center of PhenoGenomics, School of Life Sciences, Ecole Polytechnique Fédérale de Lausanne (EPFL), CH-1015 Lausanne, Switzerland; florian.lang@epfl.ch (F.L.); guillaume.valentin@epfl.ch (G.V.); 3 Zebrafish Neurogenetics Unit, Institut Pasteur, UMR3738, CNRS, 75015 Paris, France; sebastien.bedu@pasteur.fr; 4 Electro Fishing Services Ltd., Donaghadee BT21 0LN, UK; robin.mckimm@btconnect.com; 5 Cellular Biology, Physiology and Immunology Department, Universitat Autònoma de Barcelona, 08193 Bellaterra, Spain; juanramoblas@gmail.com; 6 The Francis Crick Institute, London NW1 1AT, UK; yolanda.saavedra@crick.ac.uk (Y.S.T.); sarah.wheatley@crick.ac.uk (S.E.W.); joe.higgins@crick.ac.uk (J.H.); mollie.millington@crick.ac.uk (M.E.M.); 7 Department of Biomedical sciences, Faculty of Health and Medical sciences, University of Copenhagen, 1017 Copenhagen, Denmark; plundegaard@sund.ku.dk; 8 Instituto de Investigaciones Marinas, Consejo Superior de Investigaciones Científicas, 36208 Vigo, Spain; ruchaval@iim.csic.es; 9 Institute of Pathology, Wild Animals, Fish and Bees, University of Ljubljana-Veterinary Faculty, 1000 Ljubljana, Slovenia; vlasta.jencic@vf.uni-lj.si; 10 Von Krogh Consult, 1266 Oslo, Norway; kristine.von.krogh@gmail.com

**Keywords:** zebrafish, *Danio rerio*, euthanasia, anesthesia, lidocaine, tricaine, benzocaine, clove oil, hypothermic shock, electrical stunning

## Abstract

**Simple Summary:**

Zebrafish (*Danio rerio*) younger than five days post fertilization are not protected by legislation, and protocols for their euthanasia are poorly explored. In the present paper, we assess the euthanasic efficacy of anesthetic overdose, hypothermic shock, and electrical stunning on zebrafish at <12 h post fertilization, and 1 and 4 days post fertilization utilizing laboratories in different countries. Based on the survival/recovery rates 24 h after treatment, the most effective methods were an overdose with either clove oil or lidocaine with ethanol, and electrical stunning. For the oldest larvae, signs of aversion during treatment demonstrated that all the tested anesthetics, except lidocaine, induced aversive behavior. Therefore, the most suited anesthetic treatment was lidocaine hydrochloride, 1 g/L, buffered with 2 g/L of sodium bicarbonate and mixed with 50 mL/L of ethanol. Electrical stunning also euthanized embryos and larvae efficiently and without signs of aversion; this method is new and needs further assessment in other laboratories to draw firm conclusions.

**Abstract:**

Euthanasia in zebrafish (*Danio rerio*) younger than 5 days post fertilization (dpf) is poorly described in the literature, and standardized protocols are lacking, most likely because larvae not capable of independent feeding are often not protected under national legislations. We assessed the euthanasia efficacy in laboratories in different countries of a one hour anesthetic overdose immersion with buffered lidocaine hydrochloride (1 g/L, with or without 50 mL/L of ethanol), buffered tricaine (1 g/L), clove oil (0.1%), benzocaine (1 g/L), or 2-phenoxyethanol (3 mL/L), as well as the efficacy of hypothermic shock (one hour immersion) and electrical stunning (for one minute), on zebrafish at <12 h post fertilization (hpf), 24 hpf, and 4 dpf. Based on the survival/recovery rates 24 h after treatment, the most effective methods were clove oil, lidocaine with ethanol, and electrical stunning. For 4 dpf larvae, signs of aversion during treatment demonstrated that all anesthetics, except lidocaine, induced aversive behavior. Therefore, the most suited euthanasic treatment was lidocaine hydrochloride 1 g/L, buffered with 2 g/L of sodium bicarbonate and mixed with 50 mL/L of ethanol, which euthanized both embryos and larvae in an efficient and stress-free manner. Electrical stunning also euthanized embryos and larvae efficiently and without signs of aversion; this method needs further assessment in other laboratories to draw firm conclusions.

## 1. Introduction

Under Directive 2010/63/EU, fish become protected when they reach the capacity to feed independently [1]. For zebrafish (*Danio rerio*), this is deemed to occur at about 5 days post fertilization (dpf) when the fertilized egg has been incubated at 28 °C [2]. A survey that included 145 fish laboratories in 2018 [3] revealed that the euthanasia of embryos and larvae younger than 5 dpf is mainly performed with an overdose of tricaine (also called tricaine mesylate, tricaine methanesulfonate, or MS 222). The chemical intoxication of larvae (<5 dpf), i.e., not with an anesthetic, but using substances, such as bleach or ethanol, was used by about one quarter of the respondents. Considering that larvae can respond to aversive stimuli and display other signs of consciousness similar to adults before they reach regulatory protection [4,5,6,7], the use of anesthetics should be preferred to other chemical intoxicants, for which adverse effects are less known. However, the use of, for instance, tricaine for euthanasia raises the issue of the demonstrated averseness of the compound in adult zebrafish [8,9,10,11,12,13,14]. Larvae demonstrate more resistance to a tricaine overdose than fry or adults [8,15]. A tricaine overdose should therefore be based on larval sensitivity, rather than copying adult protocols. An exploration of the efficacy of other anesthetics for embryonic and larval overdose might identify suitable alternatives to tricaine.

While electrical stunning is a euthanasia method approved by the Directive 2010/63/EU [1] and is used for the euthanasia of larger fish [16,17,18,19,20], it is not commonly used in zebrafish laboratories [3,9]. This is the opposite to hypothermic shock, a method used by many facilities for the euthanasia of zebrafish [3,9], but not mentioned in the European Directive. It is, however, recommended by the American Veterinary Medical Association guidelines for small tropical fishes [21]. Though hypothermic shock seems an efficient method for adult fish, results have been less promising for earlier developmental stages [22], with larvae <4 dpf showing close to 100% recovery after as much as 60 min in ice slurry.

It is not known how many zebrafish embryos and larvae are utilized every year, but the available data for adults indicate that numbers are high [23,24]. Combined with the knowledge that active avoidance behavior has been documented in larvae already at 72 h post fertilization (hpf) [25], and that 5 dpf larvae can display aversion and nociception much like adults [5,6,7], there is a need to develop humane euthanasia protocols for the younger life stages. To preserve animal welfare and perform ethically sound research, we must aim to euthanize by inducing a “good death”, avoiding stress, suffering, or pain. An ill-performed procedure would induce unnecessary harm and compromise animal welfare, data quality, and reproducibility. Euthanasia should induce the least distress possible in a controlled manner; it should result in either a fast loss of consciousness possibly spoiled by transitory averseness, or a slow but stress-free experience [14].

In the present study, we aim to assess whether the data obtained for the adults [26] would allow us to identify an anesthetic overdose formula that can be applied to zebrafish embryos (<12 hpf and 24 hpf) and larvae (4 dpf). Considering the variation in larval care and husbandry conditions between laboratories [27], diversity of genetic backgrounds [28], and difficulty to assess larval mortality on an individual basis, the experimental protocol was developed to be used as a multi-site experiment, with data collected from several zebrafish facilities. To explore the alternatives to overdose with known anesthetics, we also assess the efficacy of hypothermic shock and electrical stunning for zebrafish embryo and larva euthanasia.

## 2. Materials and Methods

### 2.1. Animals and Husbandry

Zebrafish embryos and larvae (wild-type on mostly AB background, see Supplementary Table S1) were used before they reached a protected developmental stage and require the authorization of the Ethics Committee. The experiments were carried out by six European zebrafish facilities listed in Table 1. Prior to each experiment, system water parameters, such as pH, temperature (°C), conductivity, degrees of general hardness (°GH), degrees of carbonate hardness (°KH), and levels of ammonia (NH_3_), nitrite (NO_2_^−^), and nitrate (NO_3_^−^), were recorded. In addition, husbandry information, such as light cycle, filtration, and feed, was provided (see Supplementary Table S1).

### 2.2. Experimental Design

Zebrafish embryos and larvae (<12 hpf, 24 hpf, and 4 dpf) were exposed to chemical or physical methods of euthanasia. At the start of each experiment, fertilized and healthy-looking embryos were collected. To reduce the potential effects from a pair of parents, mass spawning or mixing of clutches from different pairs was used to create a pool of embryos. From the common pool, the embryos were randomly distributed between the groups into Petri dishes containing embryo media. Information on the spawning time, type of embryo media, incubator temperature (°C), and potential light cycles during incubation can be found in Supplementary Table S1.

At least three replicate dishes were used per time point per treatment (for a visual presentation of the experimental set up, see Supplementary Figure S1). Each Petri dish contained 10–15 embryos or larvae (with a few exceptions of higher numbers, see Supplementary Table S3) and was cleaned daily (dead embryos and larvae were removed and embryo media replaced). 

After an incubation period of <12 hpf, 24 hpf, or 4 dpf, embryos and larvae were exposed to either an anesthetic overdose, electrical stunning, or hypothermic shock. The number and type of exposures varied between facilities (see Supplementary Table S2 for an overview of the treatments and dish replicates prepared by each facility). For the 4 dpf larvae, aversive behaviors during the treatment were recorded by a subset of facilities (see Supplementary Table S2). After exposure, embryos and larvae were transferred to clean media and incubated for another 24 h, before the number of surviving embryos and larvae were recorded.

Mortality was assessed for the respective time points with embryo coagulation at <36 hpf (for the <12 hpf time point), coagulation and absence of a heartbeat at 48 hpf (for the 24 hpf time point), lysis of tissue and the absence of a heartbeat at 5 dpf (for the 4 dpf time point). It was considered likely that the treated, but surviving, larvae had been developmentally affected during treatment, and they were therefore euthanized at the end of experiments as per local practice.

To assess the natural survival rate, unexposed control groups containing embryos and larvae obtained randomly from the common pool were included (*n* = 10–15 embryos or larvae per dish). The total number of fish in the control group was equal to or higher than the total number of fish at each time point and treatment, i.e., at least three control dishes were prepared for each experiment in each facility.

#### 2.2.1. Anesthetic Overdose

The anesthetics and buffer used in these experiments are listed in Table 2, while the working concentrations of the anesthetic solutions are listed in Table 3. All anesthetic solutions were prepared with fish system water, and the pH was measured before and after the addition of the anesthetic, buffer, and solvent (see Supplementary Table S3). Protocols were based on the published doses for adult zebrafish euthanasia induction [26]. Tricaine, 2-phenoxyethanol (2-PE), and lidocaine hydrochloride (HCl) were dissolved directly in water, while clove oil and benzocaine were first dissolved using a solvent (96% or absolute ethanol (EtOH), see Supplementary Table S3). Tricaine and lidocaine HCl solutions were buffered with sodium bicarbonate at a 2:1 ratio of the anesthetic concentration (see Table 3). All solutions and suspensions were mixed thoroughly just before use. Separate dishes with solvent and buffer controls were not included in these experiments. Nonetheless, similar concentrations of sodium bicarbonate and EtOH were used in different treatment groups (i.e., lidocaine HCl, lidocaine HCl with EtOH, and tricaine were buffered with 2 g/L NaHCO3; lidocaine HCl with EtOH and benzocaine were both 5% EtOH solutions). For lidocaine HCl with and without EtOH, a small group (3 dishes per treatment, plus control) were included for a 48 hpf time point assessment.

To reduce the additional dilution of the treatment solutions, as much embryo media as possible was removed from the dishes prior to addition of the anesthetic solution. The embryos and larvae were left immersed in the anesthetic solution for one hour. At the end of the exposure period, embryos were carefully rinsed with embryo media and placed in a new dish with fresh embryo media. For the 4 dpf time point, larvae were not removed from their dish. Instead, as much anesthetic solution as possible was removed, and the dish was rinsed with fresh embryo media before applying fresh embryo media for recovery. After 24 h of incubation in the fresh media, the number of surviving embryos and larvae was recorded. 

#### 2.2.2. Electrical Stunning

**Figure 1:**
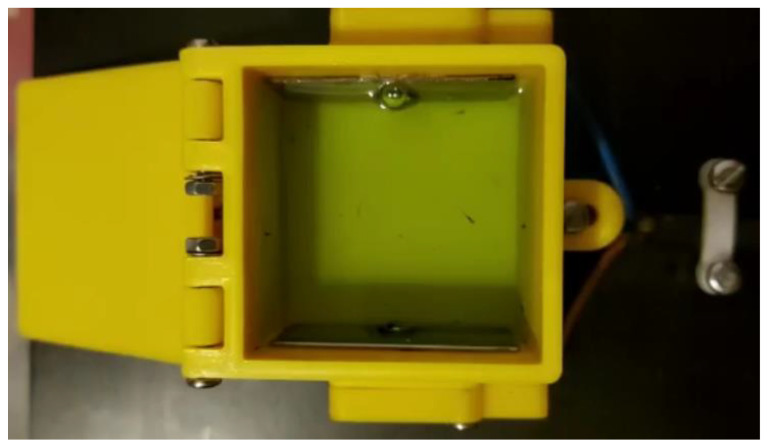
EFS-Wasp1 (Fish Management Systems Ltd., Carrickfergus, U.K.) chamber for electrical stunning with larvae (*Danio rerio*) in embryo media.

A single device (EFS-Wasp1, Fish Management Systems Ltd., U.K., see Figure 1) was purpose designed to establish whether zebrafish embryos and larvae could be euthanized reliably by electrical stunning. It was tested in only one facility on 24 hpf and 4 dpf *Danio rerio*. The device comprised a small control unit with a plug-in custom rectangular plastic specimen container fitted with full-width electrodes. A variable direct current, voltage, and pulse width were available to the electrodes. The rectangular direct current waveform had a repetition frequency of around 50 pulses per second, with a duty cycle of 50%. The used treatment voltage gradient was 25 V/cm with an electrode voltage of 100 V, and it was applied for 1 min (peak current of 0.382 amps).

Safety in use was aided by a dual button dead man’s switching system and a covering lid. The 220 V mains powered control unit had a fully isolated output, while a loud and unpleasant tone alerted the user as to when the container electrodes were live.

For the treatment, embryos and larvae were poured in their embryo media (conductivity 1.12–1.23 mS/cm) into the square (side of 4 cm) electrical stunning chamber, between the electrodes. The chamber was filled with 32 mL of embryo media to reach a depth of 2 cm, just enough to ensure full electrode immersion. After the treatment, embryos and larvae were pipetted out of the chamber, set in new embryo media, and incubated for 24 h until the number of surviving embryos and larvae was recorded. The experiments were blinded; the person who prepared and read dishes did not know treatment as the labeling system was coded. Two types of controls were used: some controls did not enter the chamber; other controls went into the chamber before and after electrical stunning for at least one minute, without undergoing a stun.

#### 2.2.3. Hypothermic Shock

The assessment of hypothermic shock as a suitable euthanasia method for embryos and larvae was performed by two facilities. The laboratories had different strategies to test the effect of hypothermic shock. At one facility, the embryos and larvae were transferred to a Petri dish with embryo media refrigerated to a temperature of 4 °C. The dish was then kept in the refrigerator at 4 °C for 1 h. At the other facility, embryo media from the original dish was removed as much as possible before chilled embryo media (1 °C) was added to the dish and the base of the dish set on the ice-chilled water (1 °C) for 1 h. The dishes were set at a level that ensured continuous chilled temperature and prevented escape. After exposure, the fish were transferred to fresh embryo media (28 °C) and incubated for 24 h, when the number of surviving embryos and larvae was recorded. The methods were considered to be so similar that the data were pooled from both experiments.

#### 2.2.4. Monitoring of Discomfort

Embryos and larvae might show signs of excitement following exposure to chemicals or physical challenges, which can be interpreted as aversion towards the events. For 4 dpf larvae, signs of aversive behavior (e.g., erratic movement and fast swimming along the edge of the Petri dish) were monitored and recorded during exposure to anesthetic overdoses (by two facilities), electrical stunning (by one facility), and hypothermic shock (by one facility).

### 2.3. Statistics and Data Presentation

Every treatment dish consisted of 10–15 embryos or larvae randomly obtained from the common pools. Because of the potential impact on each other and the embryo media parameters, individual embryos and larvae were considered pseudo-replicates for the statistical analysis. However, since embryos were randomly distributed between the dishes and treatments, each Petri dish was considered suitable as an experimental unit. Despite the single environmental condition for each pool, the multiplication of the facilities and spawning days may mitigate the influence of potential environmental effects. A total of 1 facility reported data per time point for a group of 3 dishes (e.g., for 45 fishes, see Supplementary Table S3 for details), and the average per dish was extrapolated for these values. Some experiments were performed blinded (see Supplementary Table S3 for details), and the facilities were not aware of the other facilities’ results. 

For the initial statistical analysis, each dish was scored either to have passed or failed the treatment. The dishes that “passed” had a survival/recovery rate of 0%, whereas “failed” dishes included all the dishes with at least 1 surviving larva. As we sought a treatment that works on all stages of embryos and larvae, the results from all three time points (<12 hpf, 24 hpf, and 4 dpf) were pooled when determining the treatment that was most efficient. Only the successful treatments, i.e., where more dishes passed than failed, were included in the statistical analysis. Data from the electrical stunning, although defined as a successful treatment for euthanasia, was not included in the statistical analysis as this was a pilot study at a single site and included only two time points. Additionally, due to few replicates at a single site, the 48 hpf time point for lidocaine HCl treatments was excluded from the statistical analysis. Statistically significant relationships between the treatments and survival (pass/fail) were assessed with the chi-squared test using frequencies. Post hoc tests were performed by analyzing 2 × 2 contingency tables (Fisher’s exact test) of the data, with Bonferroni adjusted significance levels. If not otherwise stated, a *p*-value of <0.05 was considered as statistically significant. The results from the statistical analysis are presented in Figure 5. However, note that the data in Figures 3–5 are presented as percent survival per dish. These figures are meant to provide the reader with more insight into the results, such as the variation in recovery rates, but no statistical analysis was performed on the continuous data. The average recovery rates were calculated, and the values are presented in the text, but not indicated in the figures. The comparison of the treatment efficacies between the lidocaine HCl with EtOH and clove oil at individual developmental stages was assessed using Fisher’s exact test (on pass/fail frequencies), with Bonferroni adjusted significance levels.

## 3. Results

**Figure 2:**
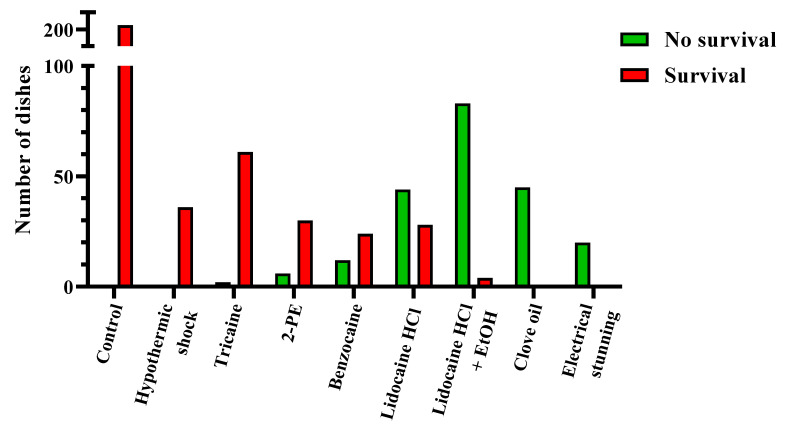
Results for zebrafish embryo and larva euthanasia, presented per experimental dish (results from <12 h post fertilization, 24 h post fertilization, and 4 days post fertilization pooled, *n* = 20–202). The dishes in which treatments lead to no surviving embryo or larva were scored as “passed” (green), whereas the dishes with one-or-more surviving embryos or larvae were scored as “failed” (red). For the results per individual time point, see Table 4.

**Figure 3:**
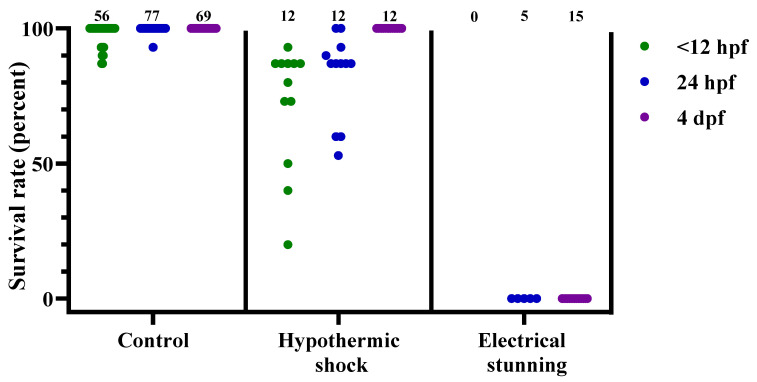
Survival rates for zebrafish following the control treatment, hypothermic shock, or electrical stunning, at 3 different developmental stages (<12 hpf, 24 hpf, and 4 dpf), presented as result per dish. Each dot represents one dish, with the total number of dishes per group indicated at the top of each column (*n* = 0–77). hpf; hours post fertilization. dpf; days post fertilization.

**Figure 4:**
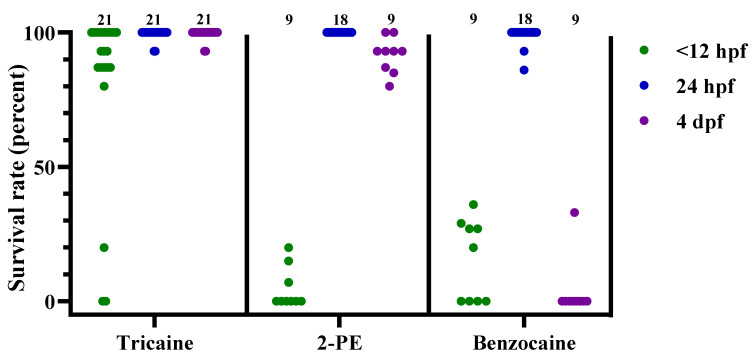
Survival rates for zebrafish following tricaine, 2-phenoxyethanol (2-PE), or benzocaine treatment, at 3 different developmental stages (<12 hpf, 24 hpf, and 4 dpf), presented as result per dish. Each dot represents one dish, with the total number of dishes per group indicated at the top of each column (*n* = 9–21). hpf; hours post fertilization. dpf; days post fertilization.

**Figure 5:**
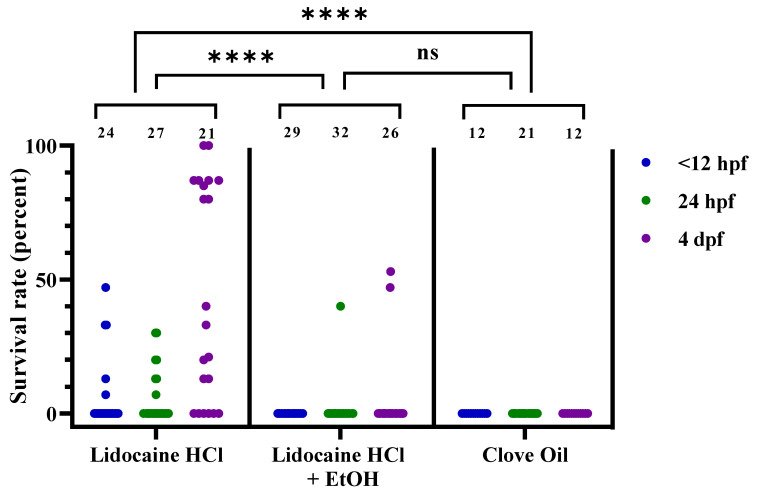
Survival rates for zebrafish following lidocaine hydrochloride (HCl), lidocaine HCl with ethanol (EtOH), or clove oil treatment, at 3 different developmental stages (<12 hpf, 24 hpf, and 4 dpf), presented as result per dish. Each dot represents one dish, with the total number of dishes per group indicated at the top of each column (*n* = 12–32). Statistical difference between the treatments was assessed by the chi-squared test (*p* < 0.0001), with post hoc analysis using Fisher’s exact test; ****; *p* < 0.0001. Note that the statistical analyses were performed on passed/failed dish frequencies (see Table 4 for details) and not on the continuous data presented here. ns; no statistical significance (*p* = 0.2987). hpf; hours post fertilization. dpf; days post fertilization.

After collecting all the survival data, each experimental dish was scored as either passing or failing the treatment. The dishes that “passed” had no surviving embryos or larvae 24 h after treatment, while the “failed” dishes had one or more embryos or larvae surviving the treatment. The pooled results from all the time points per treatment are presented in Figure 2, whereas the results from the individual time points are presented in Table 4. In Figure 3, Figure 4 and Figure 5 and Supplementary Table S3, the data are shown as the survival/recovery rate per individual dish (in percent).

As expected, mortality in the control dishes was low. The 21 control dishes that entered the electrical stunning chamber did not suffer any mortality. Altogether, out of 205 control dishes, 12 dishes had mortality (6% of dishes), with a maximum of 2 out of 15 embryos in 1 dish dying. Among all these dishes, it was more likely that the young embryos died, compared to the older larvae: in <12 hpf embryos, 10 dishes had cases of mortality within the following 24 h; in 24 hpf embryos, 2 dishes had mortality; whereas for 48 hpf embryos (3 dishes, data not presented) and 4 dpf larvae, no mortality was observed within the following 24 h (Figure 3).

Overall, our data show that the most successful treatments for zebrafish embryo and larva euthanasia were electrical stunning, clove oil, and lidocaine HCl with EtOH, whereas the least effective treatments were hypothermic shock and tricaine (Figure 2). Electrical stunning irreversibly euthanized 100% of 24 hpf embryos (*n* = 5) and 4 dpf larvae (*n* = 15) in 1 min (Figure 3). This method was not tested on embryos < 12 hpf. The other physical method, hypothermic shock, was not able to reliably euthanize zebrafish at any embryonic or larval stage tested (Figure 3). It was slightly more effective for the < 12 hpf and 24 hpf embryos than for the 4 dpf larvae, with an average recovery of 72%, 83%, and 100%, respectively (*n* = 12 for each stage).

Similar results were observed after tricaine exposure, with an average 82%, 99%, and 99% recovery following treatment at the 3 different developmental stages (Figure 4, *n* = 21 for each stage). For 2-PE and benzocaine treatments, the exposure had more variable effects depending on the developmental stage of the fish (Figure 4). Both 2-PE and benzocaine had moderate efficacy on <12 hpf embryos, with average recovery rates of 5% and 15%, respectively (*n* = 9 for both treatments). For 24 hpf embryos, on the other hand, both treatments failed to induce euthanasia, with average recovery rates of 100% and 99%, respectively (*n* = 18 for both treatments). While 2-PE and benzocaine had comparable effects on the embryonal stages, the average recovery rates for 4 dpf larvae differed substantially, at 92% vs. 4%, respectively (*n* = 9 for both treatments).

Lidocaine HCl treatment had moderate efficacy on all 3 developmental stages, with average recovery rates of 6%, 6%, and 37% (Figure 5, *n* = 24, 27, and 21, respectively). With the addition of EtOH, however, the lidocaine HCl treatment reduced the recovery rate to 0% for <12 hpf embryos, 1% for 24 hpf embryos, and 6% for 4 dpf larvae (Figure 5, *n* = 29, 32, and 26, respectively). It should be noted here that the recovery for 24 hpf embryos was observed only in 1 dish, and that some errors in the preparation of that dish cannot be excluded. For the 4 dpf larvae, recovery was observed in three dishes prepared simultaneously. These 3 dishes had a pH of 7.3, compared to pH 7.7–8.2 for the rest of the lidocaine HCl with EtOH-treated dishes (for pH levels of individual dishes, see Supplementary Table S3). Clove oil-treated embryos and larvae had 0% recovery at all stages (Figure 5, *n* = 12, 21, and 12). Statistical analysis demonstrated that the survival/recovery rate was highly dependent on treatment (chi-squared test, using pass/fail frequencies; *p* < 0.0001). Post hoc tests (Bonferroni adjusted Fisher’s exact tests) revealed lidocaine HCl to be a significantly less effective euthanasia treatment than both the lidocaine HCl with EtOH and clove oil (*p* < 0.0001). A comparison of the individual time points between the buffered lidocaine HCl with EtOH and clove oil was performed using Fisher’s exact test. No statistical difference was found between the lidocaine HCl with EtOH and clove oil efficacy at any of the three developmental stages. For both the lidocaine HCl treatments (with or without EtOH), 3 dishes were included at 48 hpf (data not presented). The average recovery rates were 13% and 0% for the lidocaine HCl without or with EtOH, respectively. Due to the low number of replicates, this time point was not included in the statistical analysis.

**Figure 6:**
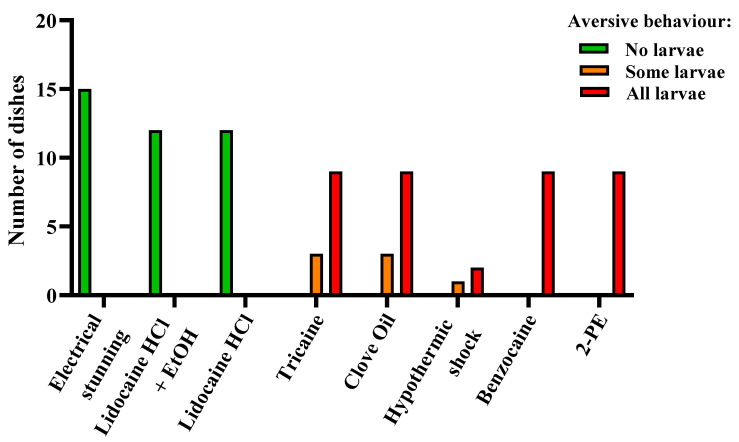
Aversive behavior in zebrafish larvae (4 days post fertilization) during electrical stunning, anesthetic overdose, and hypothermic shock, presented as result per dish. Each experimental dish (*n* = 3–15 per treatment) contained 10–15 larvae. Dishes were scored as “No larvae”, “Some larvae”, or “All larvae” depending on the proportion of the larvae displaying aversive behavior.

For a subset of the experiments involving 4 dpf larvae, monitoring for aversive behaviors during exposure to anesthetic overdoses, electrical stunning, and hypothermic shock was included. Signs of aversion were reported as and increased speed of movement, rapid directional changes, burst swimming upon treatment, or escape-like behavior, with larvae swimming at an increased speed along the rim of the dish. The treatments causing the least distress were electrical stunning and both lidocaine HCl treatments (with or without EtOH), with no larva displaying aversive behavior (Figure 6, *n* = 15 for electrical stunning and *n* = 12 for both lidocaine HCl treatments). The most distressing treatments were benzocaine and 2-PE, with all larvae showing signs of aversion (Figure 6, *n* = 9 for both treatments). For the tricaine and clove oil treatments, distress was observed in all the dishes, ranging from some larvae per dish to all larvae (Figure 6, *n* = 12 for both treatments). For hypothermic shock, the results suggest that it is an aversive treatment for zebrafish larvae, however the number of replicates is low (Figure 6, *n* = 3).

## 4. Discussion

Zebrafish embryos and larvae are widely used as research model organisms. As such, good practice of euthanasia should consider both the sample quality and fish welfare. The present study aimed at comparing the efficacy and reliability of commonly used anesthetics, hypothermic shock, and electrical stunning for zebrafish embryo and larva euthanasia using data generated in several fish facilities.

### 4.1. Anesthetic Overdoses

The natural mortality rate measured in the control dishes was low. Therefore, the lack of recovery after treatment can generally be attributed to the treatment itself and not to natural causes. The least effective treatment for the euthanasia of zebrafish embryos and larvae by overdose was buffered tricaine, with recovery rates after one hour immersion being close to that of control. This is particularly relevant considering that tricaine is widely used as a euthanasic agent for all developmental stages of fish [3,9,14,29]. However, similar results have been reported in other studies. In 9–16 dpf fry, 250 mg/L tricaine was not able to cause the cessation of a heartbeat [8]. In 14 dpf fry, cessation of a heartbeat was detected after 10 min immersion in 0.9 g/L tricaine, but even though the fry were kept immersed for an additional 20 min, there was a 100% recovery after the transfer to non-medicated water [30]. Rombough and colleagues [15] reported median LC50 (concentration that causes the death of 50% of the animals) values after one hour of immersion to be close to twice as high as the concentration used in the present study (~1.6 g/L for 3 dpf larvae and ~1.8 g/L for 4 dpf larvae vs. 1 g/L). This indicates that a higher dose than the one administrated in this study would be needed to induce the death of larvae <5 dpf. However, in all experimental dishes monitored for behavior, we found that some or all 4 dpf larvae showed signs of aversion during tricaine exposure. While increasing the dose further may alter the distress level, it is also possible that a more rapid anesthesia induction speed may mask aversive behavior display. Combining the aversive behavior reported during tricaine immersion in studies of larvae and adult zebrafish [8,9,10,11,12,13] with the uncertain euthanasic efficacy of the compound for younger stages, tricaine appears unsuited for zebrafish euthanasia at any developmental stage.

Benzocaine and 2-PE were also found to be insufficient as euthanizing agents in our experiments, though their effects varied according to the developmental stage of the fish. Neither compound was able to induce death irreversibly for 24 hpf embryos. This contrasts with <12 hpf embryos, for which both treatments were quite effective. Moreover, while 2-PE treatment did not induce euthanasia in 4 dpf larvae, benzocaine was almost 100% successful at this stage. In a recent study performed on adult zebrafish, benzocaine induced euthanasia very effectively, while the effects of 2-PE were more moderate [26]. We hypothesize that alterations occurring in the embryos and larvae during this period of development explain why these compounds are efficient at one stage, but not the next [25]. There might be very different mechanisms at hand, e.g., differential drug ability to be absorbed through chorion and larval skin or the activation or inhibition of different biochemical or neurological pathways, depending on the developmental stage of organs [15]. In addition to the lack of efficacy, both benzocaine and 2-PE induced aversive behaviors in all 4 dpf larvae investigated and, hence, cannot be recommended as euthanizing agents in zebrafish embryos and larvae with the tested protocols.

The efficacy of lidocaine HCl was assessed both with and without added EtOH. Lidocaine without EtOH was moderately effective, performing better at the embryonic stages (<12 hpf and 24 hpf) than at the larval stage (4 dpf), with an average recovery rate of 6%, 6%, and 37%, respectively. This is a higher success rate than previously observed in a study from Collymore et al. [8], in which 1 g/L lidocaine HCl treatment for 1 h was not sufficient to stop the heartbeat of 9–16 dpf fry. A possible explanation for this inconsistency could be due to fry (9–16 dpf) being less sensitive to lidocaine than 4 dpf larvae, or that the solution was not buffered sufficiently. In the study described above, the pH of the lidocaine solution was buffered to 7.0–7.4, whereas in our protocols, the solutions were buffered in the range of 7.6–8.2. In a previous report from von Krogh et al. [26], it was demonstrated that the efficacy of lidocaine HCl on the induction of adult zebrafish euthanasia is pH dependent, with a pH above 7.7 being the optimal level. The likely explanation for this is that, since lidocaine HCl has a pKa of 7.75, it will be poorly absorbed through the gills in solutions with a pH below this pKa, where lidocaine is mainly present as ionized molecules. While young larvae can rely on cutaneous gas exchange and do not depend on gills for O2 uptake until 12–14 dpf [31], a low pH may affect the ability of the drug to be absorbed through the skin and chorion. The addition of 5% EtOH to the lidocaine HCl solution greatly enhanced the efficacy at all three developmental stages, with recovery detected in only 4 out of 87 dishes. In 3 of those dishes, the pH was 7.3, lower than what we established to be the optimal pH range, which probably explains the observed recovery of the larvae. In the fourth dish, while the pH level was correct, 40% of the larvae recovered. The cause of this discrepancy is not known to us. However, this accounts for less than 1% of the treated embryos and larvae, indicating that lidocaine HCl with EtOH is a reliable and effective method for euthanasia for these life stages, when buffered correctly (pH > 7.7).

These results indicate that 5% EtOH either increases the efficacy or uptake of lidocaine and/or contributes to overdose induction on its own. Regarding the former, it is possible that EtOH increases the solubility, and therefore the absorption, of the non-ionized form of lidocaine [32]. For the latter, published data indicate that 5% EtOH exposure for 1 h is not sufficient to kill all embryos and larvae [33,34]. It is worth noting that 5% EtOH was also added to the benzocaine solutions (Table 3), which were not able to irreversibly induce death at all three developmental stages. It is therefore likely that the present findings are the result of an interaction between EtOH and lidocaine. For both lidocaine HCl treatments (with and without EtOH), no aversive behavior was detected in any larva during treatment. Lidocaine HCl has previously been demonstrated to be a fast, reliable, and relatively stress-free method for euthanasia induction in adult zebrafish [26]. The protocol with buffered lidocaine HCl and EtOH seems therefore suitable for all zebrafish developmental stages.

Clove oil is an essential oil distilled from the *Syzygium aromaticum* tree, containing eugenol as the main active ingredient. In the present study, treatment with 0.1% clove oil irreversibly euthanized all exposed embryos and larvae. It is possible that these effects depend on the age (or size) of the zebrafish, as a previous study reported full recovery in 14 dpf fry exposed to 1000 µL/L of eugenol for 75 min [30]. Furthermore, 30% of 14 dpf fry recovered after >60 min 1500 µL/L of eugenol treatment. In adults, 0.1% clove oil induces euthanasia efficiently [35], though not as efficiently as lidocaine HCl [26]. We did not find any statistically significant differences in efficacy between the clove oil and the lidocaine HCl with EtOH protocols. However, where lidocaine appears to induce death with no signs of distress, aversive behavior was observed during clove oil treatment for almost all 4 dpf larvae. While some aversion has also been reported from older zebrafish during clove oil treatment [13,36], clove oil, for instance, reduced stress during euthanasia as compared to tricaine in adults [35]. 

In conclusion, lidocaine HCl with EtOH and clove oil treatments were the most effective anesthetic overdose treatments for embryo and larva euthanasia. For routine operations, such as euthanasia, reproducibility is one of the key factors to ensure a humane process. While divergent results indicate that a method has reproducibility challenges [37], the results from lidocaine HCl with EtOH and clove oil treatments were consistent between facilities, confirming the efficacy of the formulae. For both treatments, we do not know when death occurred, i.e., during the one-hour immersion or the post-treatment incubation. Future studies could explore efficacy of shorter immersion times, mortality rate and aversion with lower doses, and address the question of speed versus fish experience, i.e., whether lidocaine induces a slow but stress-free death, whereas clove oil allows a fast but stressful loss of consciousness. Further refinements should be considered in consequence. To optimize the efficacy of embryo and larva euthanasia by overdose and reduce distress, higher doses of lidocaine HCl with EtOH could be tested to ensure a constant 100% pass rate and absence of aversive behavior. Another option consists of a two-step protocol with first an induction in lidocaine HCl with EtOH followed by clove oil immersion. Due to the aversive behavior observed here, we presently advise against using clove oil for euthanasia induction in zebrafish larvae. Rather, we recommend using the buffered lidocaine HCl with EtOH protocol. 

### 4.2. Electrical Stunning

Electrical stunning is designed to induce a loss of consciousness in less than one second and is a recommended method of euthanasia in fisheries [17,18]. Despite its approval under Directive 2010/63/EU, commercialized devices to stun laboratory fish are seldom used, and the method is not commonly used in zebrafish facilities [3,9]. Therefore, a device was specifically developed for this experiment. When farmed animals are slaughtered, electrical stunning is briefly used only to allow exsanguination. Due to the small size of zebrafish, this would be impractical. Hence, the purpose-built device provides an electric current prolonged beyond the initial stun in order to induce death without exsanguination. Thus, the term electrical stunning does not reflect well the process, which could be better described as electrical euthanasia. 

For the settings, Gross et al. [38] confirm that the early development stages of fish embryos are more susceptible to electroshock than the more-developed stages, and that voltage is the most influential factor of embryo mortality, rather than conductivity. However, fish vulnerability increases with size. Due to the small surface area and diameter of ova, a much higher voltage gradient than for an adult fish is required to change the intra cellular balanced state to an unrecoverable unbalanced state. Intense electric fields of 50 Hz are already known to be detrimental to cellular structures [39]. 

Presently, embryos and larvae (24 hpf and 4 dpf) were electrically stunned for 1 min. The results indicate that this method is 100% effective for euthanasia at these stages, and the speed of action is an unneglectable practical advantage amongst others, such as a low environmental impact and anesthetic-free samples. Nonetheless, the efficacy of electrical stunning was assessed in a single facility and not for embryos <12 hpf. A further understanding of fish welfare and experience during the process would be necessary to fully recommend this method for euthanasia. Nonetheless, the lack of displayed aversive behavior suggests that if any discomfort is induced, it is likely to be very brief.

### 4.3. Hypothermic Shock

Hypothermic shock is performed by exposing the fish to a sudden change from warm water (minimum 26 °C) to ice-chilled water (0–4 °C). While this method has been reported with high success rate for adult zebrafish [12,22,29], it is not included in the Directive 2010/63/EU as a suitable method for euthanasia. Our results indicate that hypothermic shock is not an effective method for euthanasia of embryos and larvae, as most individuals recovered after transfer back to warm water. These findings agree with earlier reports. For instance, Chen et al. [40] subjected 1–31 dpf *Danio rerio* to rapid cooling for up to 1 h. Hardly any mortality was observed in <10 dpf individuals, but mortality increased with increasing age after that stage. Wallace et al. [22] showed that younger zebrafish require prolonged exposure to rapid cooling for effective euthanasia, with the required exposure time decreasing as fish age. For 3, 4, and 7 dpf *Danio rerio*, it took 12 h of treatment to ensure death. Wallace et al. [22] also reported aversive behavior in terms of twitching in <7 dpf fish (about 25% of individuals) during hypothermic shock treatment. Other signs of distress, such as piping and erratic swimming, have been reported in older zebrafish [12,22]. While some aversion was detected in the present study, behavior was only recorded in three dishes, which is too few replicates to draw any conclusion. 

In summary, hypothermic shock is an efficient method for euthanasia of >14 dpf zebrafish, while for younger *Danio rerio*, 1 h of exposure is not a sufficient amount of time to induce death irreversibly. Furthermore, the many reports of aversion call for precaution using this method, especially for larvae that need prolonged exposure.

## 5. Conclusions

A reliable and humane protocol for euthanasia of zebrafish embryos and larvae has been lacking for many years, despite the obvious aversive behavior exhibited at these developmental stages. In the present study, a humane and reliable euthanasia protocol for zebrafish embryo and larvae at <12 hpf, 24 hpf, and 4 dpf was pursued. Using several laboratories to ensure reproducibility and reduce the potential effects of experimental bias, both chemical and physical methods were assessed. Anesthetic overdoses of tricaine, benzocaine, 2-phenoxyethanol, and lidocaine HCl proved to be either not effective at one or more developmental stages or not reliable between experiments. Furthermore, apart from lidocaine HCl, these anesthetics induced aversive behavior in 4 dpf larvae. The most effective anesthetics were clove oil and lidocaine HCl with EtOH, both treatments having high efficacy at all three developmental stages. However, clove oil induced aversive behavior in larvae and is therefore not recommended as a method for zebrafish euthanasia at this stage. No aversion was observed during lidocaine HCl with EtOH treatment.

Hypothermic shock did not work as a method of euthanasia for zebrafish embryos or larvae. Furthermore, the results indicate that this method may be aversive for the larvae. On the contrary, electrical stunning successfully euthanized 100% of the exposed 24 hpf embryos and 4 dpf larvae, and signs of aversion were not detected. These data indicate that electrical stunning is a fast, anesthetic-free, and efficient method for the euthanasia of zebrafish embryos and larvae. To complete the comparison with other tested methods, a further assessment on <12 hpf embryos and replicates for all developmental stages in another facility are necessary. It is also important to explore the experience of fish enduring the electrical stun: how much pain is induced and for how long? This may lead to questions such as: is electrical stunning linked with a fast but stressful loss of consciousness, compared to a slow but stress-free anesthesia induction? Such questions ought to be addressed to recommend the most humane euthanasia method.

From our experimental data, and in the absence of other data on electrical stunning, we conclude that 1 g/L of lidocaine HCl buffered with 2 g/L of NaHCO_3_ and mixed with 50 mL/L of EtOH is the most suited method for the euthanasia of zebrafish embryos and larvae. It is reliable and reproducible when the solution is buffered correctly, and it causes no distress. Lidocaine HCl with EtOH has previously been demonstrated to be the most suitable anesthetic for adult zebrafish overdose induction too [26]. A formula that is efficient in embryos, larvae, and adults of several months of age is likely efficient at all developmental stages. Using a single formula to ensure humane euthanasia for all individuals, regardless of age, can greatly simplify laboratory protocols for zebrafish facilities. However, as for all methods of euthanasia, there is a possibility that cellular properties, gene expression or similar, may be affected by the procedure. For embryos and larvae that are intended for scientific procedures post-mortem, this should also be taken into consideration when choosing a suitable protocol. Despite the repeated success in all facilities, the lidocaine HCl with EtOH should be tested in each facility before being used routinely, and, most importantly, buffering and solution preparation must be thoroughly controlled.

## Figures and Tables

**Table 1:** Facilities involved in the study. All websites accessed on 31 March 2022.

Name:	**Location:**	**Website:**
Ecole Polytechnique Fédérale de Lausanne (EPFL)	Lausanne, Switzerland	www.epfl.ch/en/
The Francis Crick Institute (FCI)	London, United Kingdom	www.crick.ac.uk/
Institut Pasteur (IP)	Paris, France	www.pasteur.fr/en
University of Copenhagen (KU)	Copenhagen, Denmark	www.sund.ku.dk/
Instituto de Investigaciones Marinas (IIM)	Vigo, Spain	www.iim.csic.es/
Universitat Autònoma de Barcelona (UAB)	Barcelona, Spain	www.uab.cat/en/

**Table 2:** Overview of experimental anesthetics and buffer. Sigma-Aldrich (Saint-Louis, MO, USA).

Generic Name	Active Chemical	Product Name	Manufacturer
2-Phenoxyethanol (2-PE)	2-Phenoxyethanol	2-Phenoxyethanol (77699) Aqua-Sed	SIGMA-ALDRICH (U.S.A.) VETARK (Winchester, U.K.)
Benzocaine	Ethyl 4-aminobenzoate	Benzocaine (E1501)	SIGMA-ALDRICH (U.S.A.)
Clove oil	2-Methoxy-4-(prop-2-en-1-yl)phenol (i.e., eugenol) and others unidentified	Clove oil (C8392) Essential clove oil (2215 lab 122735)	SIGMA-ALDRICH (U.S.A.) SOL NATURA (Spain)
Lidocaine hydrochloride (HCl)	2-(diethylamino)-N-(2,6- dimethylphenyl)acetamide hydrochloride monohydrate	Lidocaine hydrochloride monohydrate (L5647)	SIGMA-ALDRICH (U.S.A.)
Tricaine (MS222)	Ethyl 3-aminobenzoate methanesulfonate	Ethyl 3-aminobenzoate methanesulfonate (E10521) Ethyl 3-aminobenzoate methanesulfonate salt (A5040)	SIGMA-ALDRICH (U.S.A.) SIGMA-ALDRICH (U.S.A.)
Sodium bicarbonate (NaHCO_3_)	NaHCO_3_	Sodium bicarbonate (S5761)	SIGMA-ALDRICH (U.S.A.)

**Table 3:** Formulas for the anesthetic overdoses dissolved in the fish system water.

Agent	Dose
Lidocaine hydrochloride (HCl)	1 g/L + 2 g/L NaHCO_3_
Lidocaine HCl + ethanol (EtOH)	1 g/L + 2 g/L NaHCO_3_ + 50 mL/L EtOH
Clove oil	0.1% (1:10 in EtOH, then 10 mL/L)
Tricaine	1 g/L + 2 g/L NaHCO_3_
Benzocaine	1 g /L (20 g/L in EtOH, then 50 mL/L)
2-Phenoxyethanol (2-PE)	3 mL/L

**Table 4:** Success of the experimental method for zebrafish embryo and larva euthanasia, results per dish. Passed; dishes had zero surviving embryos or larvae after treatment. Failed; dishes had one-or-more embryo or larva surviving treatment. hpf; hours post fertilization. dpf; days post fertilization.

Treatment	Timepoint	Dishes			% Passed	
		Passed	Failed	Total	Time Point	Treatment
Control	<12 hpf	0	56	56	0%	0%
	24 hpf	0	77	77	0%	
	48 h	0	3	3	0%	
	4 dpf	0	69	69	0%	
Hypothermic shock	<12 hpf	0	12	12	0%	0%
	24 hpf	0	12	12	0%	
	4 dpf	0	12	12	0%	
Tricaine	<12 hpf	2	19	21	10%	3%
	24 hpf	0	21	21	0%	
	4 dpf	0	21	21	0%	
2-phenoxyethanol	<12 hpf	6	3	9	67%	22%
	24 hpf	0	18	18	0%	
	4 dpf	0	9	9	0%	
Benzocaine	<12 hpf	4	5	9	44%	44%
	24 hpf	0	18	18	0%	
	4 dpf	8	1	9	89%	
Lidocaine HCl	<12 hpf	19	5	24	79%	61%
	24 hpf	19	8	27	70%	
	48 h	2	1	3	67%	
	4 dpf	6	15	21	29%	
Lidocaine HCL + EtOH	<12 hpf	29	0	29	100%	96%
	24 hpf	31	1	32	97%	
	48 h	3	0	3	100%	
	4 dpf	23	3	26	88%	
Clove oil	<12 hpf	12	0	12	100%	100%
	24 hpf	21	0	21	100%	
	4 dpf	12	0	12	100%	
Electrical stunning	24 hpf	5	0	5	100%	100%
	4 dpf	15	0	15	100%	

## Data Availability

Original data are available on request.

## Supplementary Materials

The following supporting information can be downloaded at: https://www.mdpi.com/article/10.3390/biology11040546/s1, Figure S1: Diagram of experimental set-up, Table S1: Husbandry conditions, Table S2: Overview of treatment and dish replicates prepared by each facility, and Table S3: Data per fish and further details on facility conditions.
