# Supplementary material for: A Multi-Site Assessment of Anesthetic Overdose, Hypothermic Shock, and Electrical Stunning as Methods of Euthanasia for Zebrafish (Danio rerio) Embryos and Larvae
Source: Biology (Basel). 2022 Apr 1;11(4):546. doi: 10.3390/biology11040546 (PMC9027676; doi:10.3390/biology11040546)
Supplement: Supplementary file 1 [file biology-11-00546-s001.zip › Supplementary Figure S1.pdf]

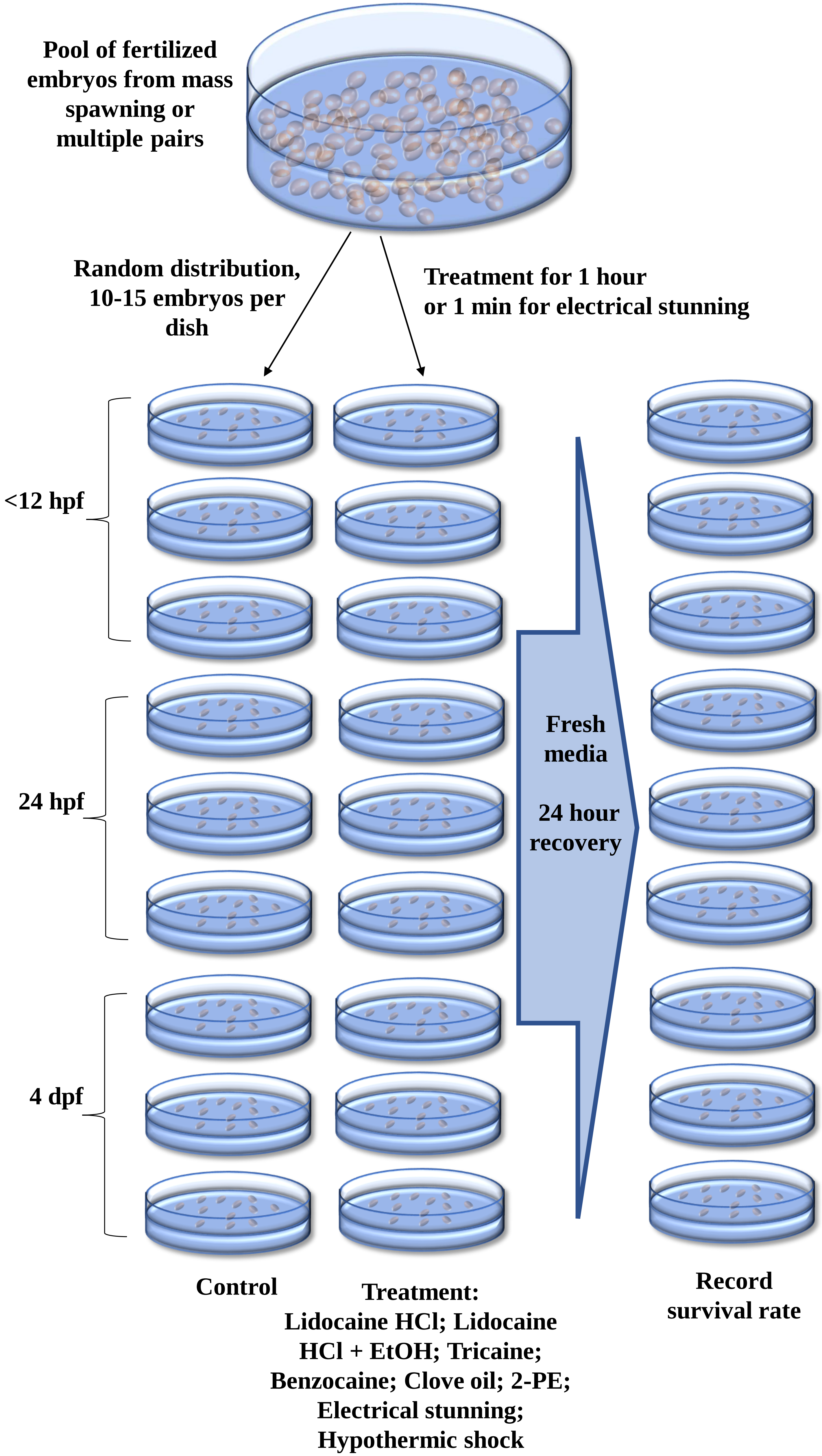
Pool of fertilized embryos from mass spawning or multiple pairs
Random distribution, 10-15 embryos per dish
Treatment for 1 hour or 1 min for electrical stunning
<12 hpf
24 hpf
4 dpf
Fresh media
24 hour recovery
Control
Treatment: Lidocaine HCl; Lidocaine HCl + EtOH; Tricaine; Benzocaine; Clove oil; 2-PE; Electrical stunning; Hypothermic shock
Record survival rate
