# Supplementary material for: A Multi-Site Assessment of Anesthetic Overdose, Hypothermic Shock, and Electrical Stunning as Methods of Euthanasia for Zebrafish (Danio rerio) Embryos and Larvae
Source: Biology (Basel). 2022 Apr 1;11(4):546. doi: 10.3390/biology11040546 (PMC9027676; doi:10.3390/biology11040546)
Supplement: Supplementary file 1 [file biology-11-00546-s001.zip › Supplementary Table S1.pdf]

Supplementary Table S1

Husbandry conditions

- Adult system water conditions per facility page 1

- Adult feeds per facility page 2

- Spawning and incubation conditions page 3

- Adult system water conditions per facility

Facility	Adult System Water											
	pH	Temperature (°C)	Conductivity (mS/cm)	Ammonia (ppm NH_3)	Nitrite (ppm NO_2^-)	Nitrate (ppm NO_3^-)	Hardness (°GH)	Alkalinity (°KH)	Light cycle (light/night)	Reverse Osmosis	Recirculation	Carbon filter on recirculation
**EPFL**	7.5-7.8	26.0	0.411-0.425	0	/	2.0-4.7	5-6	/	14h/10h	Yes	Yes	No
**FCI**	7.5-8.0	27.7-28.0	0.420-0.450	0	0	27-29	3	2-3	14h/10h	Yes	Yes	Yes
**IP**	7.1	27.7-28.4	0.520-0.532	< 0.05	0.1	20	1	2	14h/10h	Yes	Yes	Yes
**KU**	7.3	28.0-28.4	0.800-0.812	< 0.05	0.02	20	7	4	14h/10h	Yes	Yes	Yes
**IIM**	7.0	28.0	0.700	0	0	/	1	/	12h/12h	No	Yes	No
**UAB**	7.3	27.0	0.850	< 0.05	0.5	< 50	4	4	14h/10h	Yes	Yes	Yes

/; data are not available

- Adult feeds per facility

Facility	Adult Feeds	
	**List dry feed used for all stages**	**List live feed used for all stages**
**EPFL**	Sparos	Paramecia / Artemia
**FCI**	ZM, SAFE caviar	Paramecia / Artemia
**IP**	Gemma Micro	Rotifers
**KU**	ZM, SDS, and Sparos	Artemia
**IIM**	Nutrafin max	Artemia
**UAB**	Sparos	Artemia

- Spawning and incubation conditions

Most used strains were Wild Type (WT) AB. These were second generation progeny from the Zebrafish International Resource Center (ZIRC, Eugene, OR, USA) or later generations.

**Faculty**	**Mating**				**Incubator conditions**		
	**Spawning set-up**	**Time mated**	**Spawning time**	**Strain**	**Incubator's temperature (°C)**	**Embryo media**	**Light cycle during incubation (light/night)**
**EPFL**	Multiple pairs	Yes	09:30	WT AB	28.5	System water	No
**FCI**	Mass spawning	No	08:00-09:00	WT AB	28.0	0.5 X E2	No
**IP**	Multiple pairs	Yes	09:00	WT AB	28.0	E3	Yes, 14h/10h
**KU**	Multiple pairs	No	08:30	WT AB	28.5	E3	No
**IIM**	Mass spawning	Yes	09:00	WT	28.0	System water	Yes, 12h/12h
**UAB**	Multiple pairs	Yes	08:00	WT AB	28.0	In-house mix	Yes, 12h/12h

Recipes for embryo media:

FCI: 7.5 mM NaCl; 0.25 mM KCl; 0.5 mM $CaCl_2$; 0.5 mM $MgSO_4$; 75 μM KH_2PO_4; 25 μM Na_2HPO_4; 0.35 mM $NaHCO_3$; 0.5 mg/L Methylene Blue.

IP: 4.5 mM NaCl; 0.17 mM KCl; 0.4 mM $CaCl_2$.

KU: 5 mM NaCl; 0.17 mM KCl; 0.33 mM $CaCl_2$; 0.33 mM $MgSO_4$.

UAB: Distilled water; NaCl; $NaHCO_3$; pH 7.3; conductivity 0.8 +/- 0.1 mS/cm.
