# Supplementary material for: A Multi-Site Assessment of Anesthetic Overdose, Hypothermic Shock, and Electrical Stunning as Methods of Euthanasia for Zebrafish (Danio rerio) Embryos and Larvae
Source: Biology (Basel). 2022 Apr 1;11(4):546. doi: 10.3390/biology11040546 (PMC9027676; doi:10.3390/biology11040546)
Supplement: Supplementary file 1 [file biology-11-00546-s001.zip › Supplementary Table S2.pdf]

# Supplementary Table S2

Overview of treatment and dish replicates prepared by each facility

*; behaviour was assessed for these dishes.

| Facility | Pool date | Lidocaine HCl+EtOH | | | | Lidocaine HCl | | | | Tricaine | | | Clove Oil | | | Benzocaine | | | 2-phenoxyethanol | | | Electrical stunning | | | Hypothermic shock | | |
|---|---|---|---|---|---|---|---|---|---|---|---|---|---|---|---|---|---|---|---|---|---|---|---|---|---|---|---|
| | | <12hpf | 24hpf | 48hpf | 4dpf | <12hpf | 24hpf | 48hpf | 4dpf | <12hpf | 24hpf | 4dpf | <12hpf | 24hpf | 4dpf | <12hpf | 24hpf | 4dpf | <12hpf | 24hpf | 4dpf | <12hpf | 24hpf | 4dpf | <12hpf | 24hpf | 4dpf |
| EPFL | 22JUN20 | 3 | | | | 3 | | | | 3 | | | 3 | | | 3 | | | 3 | | | | | | | | |
| EPFL | 23JUN20 | | 3 | | | | 3 | | | | 3 | | | 3 | | | 3 | | | 3 | | | | | | | |
| EPFL | 01JUL20 | 3 | | | | 3 | | | | 3 | | | 3 | | | 3 | | | 3 | | | | | | | | |
| EPFL | 30JUN20 | | 3 | | | | 3 | | | | 3 | | | 3 | | | 3 | | | 3 | | | | | | | |
| EPFL | 27JUN20 | | | | 3* | | | | 3* | | | 3* | | | 3* | | | 3* | | | 3* | | | | | | |
| EPFL | 07JUL20 | 3 | | | | 3 | | | | 3 | | | 3 | | | 3 | | | 3 | | | | | | | | |
| EPFL | 06JUL20 | | 3 | | | | 3 | | | | 3 | | | 3 | | | 3 | | | 3 | | | | | | | |
| EPFL | 03JUL20 | | | | 3* | | | | 3* | | | 3* | | | 3* | | | 3* | | | 3* | | | | | | |
| EPFL | 26AUG20 | | | | 3* | | | | 3* | | | 3* | | | 3* | | | 3* | | | 3* | | | | | | |
| FCI | 17NOV20 | | | | | | | | | | | | | 3 | | | 3 | | | 3 | | | | | | | |
| FCI | 24NOV20 | | | | | | | | | | | | | 3 | | | 3 | | | 3 | | | | | | | |
| FCI | 01DEC20 | | | | | | | | | | | | | 3 | | | 3 | | | 3 | | | | | | | |
| FCI | 08DEC21 | | | | | | | | | | | | | | | | | | | | | | 5 | 5* | | | |
| FCI | 15DEC21 | | | | | | | | | | | | | | | | | | | | | | | 10* | | | |
| IP | 16NOV20 | 3 | 3 | | | 3 | 3 | | | 3 | 3 | | | | | | | | | | | | | | 3 | 3 | |
| IP | 23NOV20 | 3 | 3 | | 3 | 3 | 3 | | 3 | 3 | 3 | 3 | | | | | | | | | | | | | 3 | 3 | 3 |
| IP | 26NOV20 | | | | 3 | | | | 3 | | | 3 | | | | | | | | | | | | | | | 3 |
| IP | 30NOV20 | 3 | 3 | | 3 | 3 | 3 | | 3 | 3 | 3 | 3 | | | | | | | | | | | | | 3 | 3 | 3 |
| KU | 25FEB20 | 3 | 3 | 3 | | 3 | 3 | 3 | | | | | | | | | | | | | | | | | | | |
| KU | 16JUN21 | | 3 | 3 | | | 3 | 3 | | | | | | | | | | | | | | | | | | | |
| IIM | 12JAN21 | 5 | 5 | | | | | | | | | | | | | | | | | | | | | | | | |
| IIM | 14JAN21 | | | | 5 | | | | | | | | | | | | | | | | | | | | | | |
| UAB | 27OCT20 | 3 | 3 | | 3* | 3 | 3 | | 3* | 3 | 3 | 3* | 3 | 3 | 3* | | | | | | | | | | 3 | 3 | 3* |
