# Supplementary material for: A Multi-Site Assessment of Anesthetic Overdose, Hypothermic Shock, and Electrical Stunning as Methods of Euthanasia for Zebrafish (Danio rerio) Embryos and Larvae
Source: Biology (Basel). 2022 Apr 1;11(4):546. doi: 10.3390/biology11040546 (PMC9027676; doi:10.3390/biology11040546)
Supplement: Supplementary file 1 [file biology-11-00546-s001.zip › Supplementary Table S3.pdf]

# Supplementary Table S3

## Ethanol used in each facility

| | | |
|---|---|---|
| **EPFL** | Ethanol Absolute | Fisher Chemical E/0650DF/17, Fisher Scientific, UK |
| **FCI** | Ethanol 96% | Fisher Chemical E/0555DF/17, Fisher Scientific, UK |
| **IP** | Ethanol Absolute | 32205-M, SIGMA-ALDRICH, USA |
| **KU** | Ethanol Absolute | 1009831011, SIGMA-ALDRICH, USA |
| **IIM** | Ethanol 96% | Alcoholes Galicia S. L., Spain |
| **UAB** | Ethanol 96% | Not available |

## Blinded experiments

| | |
|---|---|
| **EPFL** | No |
| **FCI** | Not blinded for anaesthesia overdose. Blinded for electrical stunning. |
| **IP** | No |
| **KU** | No |
| **IIM** | Yes |
| **UAB** | Yes |

## Tables presenting data per fish

Data are presented per treatment, time point, facility, and pool date. Each row represents a dish (or a group of 3 dishes for IP). Each pool is identified by the date at which the pool of eggs was fertilised.

- In the third column, the number of embryos or larvae undergoing treatment is indicated.

- In the fourth column, the number of embryos or larvae that survived 24 hours post treatment is indicated.

- The fifth column indicates the percentage of embryos or larvae that survived in each dish.

- The pH of the anaesthetic solution was measured before and after anaesthetics, buffer, and solvent were added, and these data are indicated for each day of treatment.


| <u>Treatment</u> | <u>Pages</u> |
|---|---|
| Lidocaine HCl with EtOH | 2-4 |
| Lidocaine HCl | 5-7 |
| Tricaine | 8-10 |
| Clove oil | 11-12 |
| Benzocaine | 13-14 |
| 2-phenoxyethanol | 15-16 |
| Electrical stunning | 17 |
| Hypothermic shock | 18 |

# Lidocaine HCl with EtOH

## Lidocaine hydrochloride 1 g/L + sodium bicarbonate 2 g/L + 50 mL/L ethanol

### < 12 hpf Lidocaine HCl with EtOH

| Facility | Pool | <12hpf | <36hpf | % Survival | pH before | pH after |
|---|---|---|---|---|---|---|
| EPFL | 22JUN20 | 14 | 0 | 0% | 7.7 | 7.7 |
| | | 15 | 0 | 0% | | |
| | | 15 | 0 | 0% | | |
| | 01JUL20 | 15 | 0 | 0% | 7.6 | 7.8 |
| | | 15 | 0 | 0% | | |
| | | 15 | 0 | 0% | | |
| | 07JUL20 | 15 | 0 | 0% | 7.5 | 7.7 |
| | | 15 | 0 | 0% | | |
| | | 15 | 0 | 0% | | |
| IP | 16NOV20 | 45 | 0 | 0% | 7.3 | 7.7 |
| | 23NOV20 | 45 | 0 | 0% | 7.3 | 7.7 |
| | 30NOV20 | 45 | 0 | 0% | 7.3 | 7.7 |
| KU | 25FEB20 | 10 | 0 | 0% | 7.6 | 8.2 |
| | | 10 | 0 | 0% | | |
| | | 10 | 0 | 0% | | |
| IIM | 12JAN21 | 15 | 0 | 0% | Not measured | Not measured |
| | | 15 | 0 | 0% | | |
| | | 15 | 0 | 0% | | |
| | | 15 | 0 | 0% | | |
| | | 15 | 0 | 0% | | |
| UAB | 27OCT20 | 10 | 0 | 0% | 7.6 | 7.8 |
| | | 10 | 0 | 0% | | |
| | | 10 | 0 | 0% | | |

**24 hpf Lidocaine HCl with EtOH**

| Facility | Pool | 24hpf | 48hpf | % Survival | pH before | pH after |
|---|---|---|---|---|---|---|
| EPFL | 23JUN20 | 15 | 0 | 0% | 7.7 | 7.8 |
| | | 14 | 0 | 0% | | |
| | | 13 | 0 | 0% | | |
| | 30JUN20 | 15 | 0 | 0% | 7.6 | 7.7 |
| | | 15 | 0 | 0% | | |
| | | 15 | 0 | 0% | | |
| | 06JUL20 | 15 | 0 | 0% | 7.5 | 7.7 |
| | | 15 | 0 | 0% | | |
| | | 15 | 0 | 0% | | |
| IP | 16NOV20 | 45 | 0 | 0% | 7.3 | 7.7 |
| | 23NOV20 | 45 | 0 | 0% | 7.3 | 7.7 |
| | 30NOV20 | 45 | 0 | 0% | 7.3 | 7.7 |
| KU | 25FEB20 | 10 | 0 | 0% | 7.6 | 8.2 |
| | | 10 | 4 | 40% | | |
| | | 10 | 0 | 0% | | |
| | 16JUN21 | 10 | 0 | 0% | 7.5 | 7.9 |
| | | 10 | 0 | 0% | | |
| | | 10 | 0 | 0% | | |
| IIM | 12JAN21 | 15 | 0 | 0% | Not measured | Not measured |
| | | 15 | 0 | 0% | | |
| | | 15 | 0 | 0% | | |
| | | 15 | 0 | 0% | | |
| | | 15 | 0 | 0% | | |
| UAB | 27OCT20 | 10 | 0 | 0% | 7.5 | 7.7 |
| | | 10 | 0 | 0% | | |
| | | 10 | 0 | 0% | | |

**48 hpf Lidocaine HCl with EtOH**

| Facility | Pool | 48hpf | 72hpf | % Survival | pH before | pH after |
|---|---|---|---|---|---|---|
| KU | 16JUN21 | 10 | 0 | 0% | 7.5 | 8.0 |
| | | 10 | 0 | 0% | | |
| | | 10 | 0 | 0% | | |

**4 dpf Lidocaine HCl with EtOH**

| Facility | Pool | 4dpf | 5dpf | % Survival | pH before | pH after |
|---|---|---|---|---|---|---|
| EPFL | 27JUN20 | 15 | 0 | 0% | 7.6 | 7.7 |
| | | 15 | 0 | 0% | | |
| | | 15 | 0 | 0% | | |
| | 03JUL20 | 15 | 0 | 0% | 7.5 | 7.8 |
| | | 15 | 0 | 0% | | |
| | | 15 | 0 | 0% | | |
| | 26AUG20 | 15 | 0 | 0% | 7.6 | 7.9 |
| | | 15 | 0 | 0% | | |
| | | 15 | 0 | 0% | | |
| IP | 26NOV20 | 41 | 0 | 0% | 7.3 | 7.7 |
| | 23NOV20 | 45 | 0 | 0% | 7.2 | 7.7 |
| | 30NOV20 | 45 | 22 | 49% | 7.2 | 7.3 |
| IIM | 14JAN21 | 15 | 0 | 0% | Not measured | Not measured |
| | | 15 | 0 | 0% | | |
| | | 15 | 0 | 0% | | |
| | | 15 | 0 | 0% | | |
| | | 15 | 0 | 0% | | |
| UAB | 27OCT20 | 10 | 0 | 0% | 7.6 | 7.8 |
| | | 10 | 0 | 0% | | |
| | | 10 | 0 | 0% | | |

# Lidocaine HCl

## Lidocaine hydrochloride 1 g/L + sodium bicarbonate 2 g/L

### < 12 hpf Lidocaine HCl

| Facility | Pool | <12hpf | <36hpf | % Survival | pH before | pH after |
|---|---|---|---|---|---|---|
| **EPFL** | 22JUN20 | 15 | 0 | 0% | 7.7 | 7.7 |
| | | 15 | 0 | 0% | | |
| | | 15 | 0 | 0% | | |
| | 01JUL20 | 15 | 0 | 0% | 7.6 | 7.8 |
| | | 15 | 0 | 0% | | |
| | | 15 | 0 | 0% | | |
| | 07JUL20 | 15 | 0 | 0% | 7.5 | 7.7 |
| | | 15 | 1 | 7% | | |
| | | 15 | 2 | 13% | | |
| **IP** | 16NOV20 | 45 | 0 | 0% | 7.3 | 7.6 |
| | 23NOV20 | 45 | 0 | 0% | 7.3 | 7.6 |
| | 30NOV20 | 45 | 17 | 38% | 7.3 | 7.6 |
| **KU** | 25FEB20 | 10 | 0 | 0% | 7.6 | 8.2 |
| | | 10 | 0 | 0% | | |
| | | 10 | 0 | 0% | | |
| **UAB** | 27OCT20 | 10 | 0 | 0% | 7.7 | 7.9 |
| | | 10 | 0 | 0% | | |
| | | 10 | 0 | 0% | | |

**24 hpf Lidocaine HCl**

| Facility | Pool | 24hpf | 48hpf | % Survival | pH before | pH after |
|---|---|---|---|---|---|---|
| EPFL | 23JUN20 | 15 | 0 | 0% | 7.7 | 7.7 |
| | | 15 | 0 | 0% | | |
| | | 15 | 0 | 0% | | |
| | 30JUN20 | 14 | 0 | 0% | 7.6 | 7.7 |
| | | 15 | 0 | 0% | | |
| | | 15 | 0 | 0% | | |
| | 06JUL20 | 15 | 0 | 0% | 7.5 | 7.7 |
| | | 15 | 0 | 0% | | |
| | | 15 | 0 | 0% | | |
| IP | 16NOV20 | 45 | 5 | 11% | 7.3 | 7.6 |
| | 23NOV20 | 45 | 0 | 0% | 7.3 | 7.6 |
| | 30NOV20 | 45 | 0 | 0% | 7.3 | 7.6 |
| KU | 25FEB20 | 10 | 3 | 30% | 7.6 | 8.2 |
| | | 10 | 3 | 30% | | |
| | | 10 | 2 | 20% | | |
| | 16JUN21 | 10 | 0 | 0% | 7.5 | 8.0 |
| | | 10 | 2 | 20% | | |
| | | 10 | 4 | 40% | | |
| UAB | 27OCT20 | 10 | 0 | 0% | 7.6 | 7.8 |
| | | 10 | 0 | 0% | | |
| | | 10 | 0 | 0% | | |

**48 hpf Lidocaine HCl**

| Facility | Pool | 48hpf | 72hpf | % Survival | pH before | pH after |
|---|---|---|---|---|---|---|
| KU | 16JUN21 | 10 | 0 | 0% | 7.6 | 8.0 |
| | | 10 | 4 | 40% | | |
| | | 10 | 0 | 0% | | |

**4 dpf Lidocaine HCl**

| Facility | Pool | 4dpf | 5dpf | % Survival | pH before | pH after |
|---|---|---|---|---|---|---|
| **EPFL** | 27JUN20 | 15 | 5 | 33% | 7.6 | 7.7 |
| | | 15 | 6 | 40% | | |
| | | 15 | 2 | 13% | | |
| | 03JUL20 | 14 | 3 | 21% | 7.5 | 7.8 |
| | | 15 | 2 | 13% | | |
| | | 15 | 0 | 0% | | |
| | 26AUG20 | 15 | 3 | 20% | 7.6 | 7.9 |
| | | 15 | 0 | 0% | | |
| | | 14 | 0 | 0% | | |
| **IP** | 26NOV20 | 43 | 35 | 81% | 7.3 | 7.6 |
| | 23NOV20 | 45 | 39 | 87% | 7.2 | 7.6 |
| | 30NOV20 | 45 | 43 | 96% | 7.2 | 7.6 |
| **UAB** | 27OCT20 | 10 | 0 | 0% | 7.6 | 7.8 |
| | | 10 | 0 | 0% | | |
| | | 10 | 0 | 0% | | |

# Tricaine

## Tricaine 1 g/L with 2 g/L Sodium bicarbonate

**EPFL and IP:** Tricaine, Ethyl 3-aminobenzoate methanesulfonate salt, Sigma-aldrich, A5040, USA

**UAB:** Tricaine, Ethyl 3-aminobenzoate methanesulfonate, Sigma-aldrich, E10521, USA

### < 12 hpf Tricaine

| Facility | Pool | <12hpf | <36hpf | % Survival | pH before | pH after |
|---|---|---|---|---|---|---|
| **EPFL** | 22JUN20 | 15 | 14 | 93% | 7.7 | 7.7 |
| | | 15 | 13 | 87% | | |
| | | 15 | 15 | 100% | | |
| | 01JUL20 | 15 | 15 | 100% | 7.6 | 7.8 |
| | | 15 | 15 | 100% | | |
| | | 15 | 15 | 100% | | |
| | 07JUL20 | 15 | 15 | 100% | 7.5 | 7.7 |
| | | 15 | 15 | 100% | | |
| | | 15 | 15 | 100% | | |
| **IP** | 16NOV20 | 45 | 38 | 84% | 7.3 | 7.1 |
| | 23NOV20 | 45 | 40 | 89% | 7.3 | 7.1 |
| | 30NOV20 | 45 | 44 | 98% | 7.3 | 7.2 |
| **UAB** | 27OCT20 | 10 | 0 | 0% | 7.6 | 7.6 |
| | | 10 | 0 | 0% | | |
| | | 10 | 2 | 20% | | |

**24 hpf Tricaine**

| Facility | Pool | 24hpf | 48hpf | % Survival | pH before | pH after |
|---|---|---|---|---|---|---|
| **EPFL** | 23JUN20 | 15 | 15 | 100% | 7.7 | 7.8 |
| | | 15 | 15 | 100% | | |
| | | 15 | 15 | 100% | | |
| | 30JUN20 | 15 | 15 | 100% | 7.6 | 7.7 |
| | | 15 | 15 | 100% | | |
| | | 15 | 15 | 100% | | |
| | 06JUL20 | 15 | 15 | 100% | 7.5 | 7.7 |
| | | 15 | 15 | 100% | | |
| | | 15 | 15 | 100% | | |
| **IP** | 16NOV20 | 45 | 43 | 96% | 7.3 | 7.1 |
| | 23NOV20 | 45 | 45 | 100% | 7.3 | 7.1 |
| | 30NOV20 | 44 | 44 | 100% | 7.3 | 7.2 |
| **UAB** | 27OCT20 | 10 | 10 | 100% | 7.6 | 7.6 |
| | | 10 | 10 | 100% | | |
| | | 10 | 10 | 100% | | |

**4 dpf Tricaine**

| Facility | Pool | 4dpf | 5dpf | % Survival | pH before | pH after |
|---|---|---|---|---|---|---|
| **EPFL** | 27JUN20 | 15 | 15 | 100% | 7.6 | 7.7 |
| | | 15 | 15 | 100% | | |
| | | 15 | 15 | 100% | | |
| | 03JUL20 | 15 | 15 | 100% | 7.5 | 7.7 |
| | | 15 | 15 | 100% | | |
| | | 15 | 14 | 93% | | |
| | 26AUG20 | 15 | 15 | 100% | 7.6 | 7.9 |
| | | 13 | 13 | 100% | | |
| | | 15 | 15 | 100% | | |
| **IP** | 26NOV20 | 45 | 45 | 100% | 7.3 | 7.2 |
| | 23NOV20 | 45 | 45 | 100% | 7.2 | 7.1 |
| | 30NOV20 | 44 | 43 | 98% | 7.2 | 7.1 |
| **UAB** | 27OCT20 | 10 | 10 | 100% | 7.7 | 7.6 |
| | | 10 | 10 | 100% | | |
| | | 10 | 10 | 100% | | |

# Clove oil 0.1%

## 1:10 clove oil in ethanol, then 10 mL/L

**EPFL and FCI:** Clove Oil, Sigma-Aldrich, C8392, USA

**UAB:** Essential clove oil, Sol Natura, 2,215 lab 122735, Spain

### < 12 hpf Clove oil

| Facility | Pool | <12hpf | <36hpf | % Survival | pH before | pH after |
|---|---|---|---|---|---|---|
| | | 13 | 0 | 0% | | |
| | 22JUN20 | 14 | 0 | 0% | 7.7 | 7.4 |
| | | 10 | 0 | 0% | | |
| | | 15 | 0 | 0% | | |
| **EPFL** | 01JUL20 | 15 | 0 | 0% | 7.6 | 7.4 |
| | | 15 | 0 | 0% | | |
| | | 15 | 0 | 0% | | |
| | 07JUL20 | 15 | 0 | 0% | 7.5 | 7.6 |
| | | 15 | 0 | 0% | | |
| | | 10 | 0 | 0% | | |
| **UAB** | 27OCT20 | 10 | 0 | 0% | Not measured | Not measured |
| | | 10 | 0 | 0% | | |

**24 hpf Clove oil**

| Facility | Pool | 24hpf | 48hpf | % Survival | pH before | pH after |
|---|---|---|---|---|---|---|
| EPFL | 23JUN20 | 15 | 0 | 0% | 7.7 | 7.5 |
| | | 14 | 0 | 0% | | |
| | | 15 | 0 | 0% | | |
| | 30JUN20 | 12 | 0 | 0% | 7.6 | 7.6 |
| | | 15 | 0 | 0% | | |
| | | 13 | 0 | 0% | | |
| | 06JUL20 | 15 | 0 | 0% | 7.5 | 7.4 |
| | | 15 | 0 | 0% | | |
| | | 15 | 0 | 0% | | |
| FCI | 17NOV20 | 15 | 0 | 0% | 7.0 | 7.2 |
| | | 15 | 0 | 0% | | |
| | | 15 | 0 | 0% | | |
| | 24NOV20 | 15 | 0 | 0% | 6.8 | 7.1 |
| | | 15 | 0 | 0% | | |
| | | 15 | 0 | 0% | | |
| | 01DEC20 | 10 | 0 | 0% | 6.7 | 7.4 |
| | | 10 | 0 | 0% | | |
| | | 15 | 0 | 0% | | |
| UAB | 27OCT20 | 10 | 0 | 0% | Not measured | Not measured |
| | | 10 | 0 | 0% | | |
| | | 10 | 0 | 0% | | |

**4 dpf Clove oil**

| Facility | Pool | 4dpf | 5dpf | % Survival | pH before | pH after |
|---|---|---|---|---|---|---|
| EPFL | 27JUN20 | 15 | 0 | 0% | 7.6 | 7.5 |
| | | 15 | 0 | 0% | | |
| | | 15 | 0 | 0% | | |
| | 03JUL20 | 15 | 0 | 0% | 7.5 | 7.4 |
| | | 15 | 0 | 0% | | |
| | | 15 | 0 | 0% | | |
| | 26AUG20 | 15 | 0 | 0% | 7.6 | 7.1 |
| | | 15 | 0 | 0% | | |
| | | 15 | 0 | 0% | | |
| UAB | 27OCT20 | 10 | 0 | 0% | Not measured | Not measured |
| | | 10 | 0 | 0% | | |
| | | 10 | 0 | 0% | | |

# Benzocaine 1g/L

## 20 g/L in ethanol, then 50 mL/L

### < 12 hpf Benzocaine

| Facility | Pool | <12hpf | <36hpf | % Survival | pH before | pH after |
|---|---|---|---|---|---|---|
| EPFL | 22JUN20 | 15 | 4 | 27% | 7.7 | 7.6 |
| | | 14 | 4 | 29% | | |
| | | 15 | 4 | 27% | | |
| | 01JUL20 | 15 | 0 | 0% | 7.6 | 7.6 |
| | | 14 | 0 | 0% | | |
| | | 15 | 0 | 0% | | |
| | 07JUL20 | 15 | 0 | 0% | 7.5 | 7.7 |
| | | 14 | 5 | 36% | | |
| | | 15 | 3 | 20% | | |

### 24 hpf Benzocaine

| Facility | Pool | 24hpf | 48hpf | % Survival | pH before | pH after |
|---|---|---|---|---|---|---|
| EPFL | 23JUN20 | 15 | 15 | 100% | 7.7 | 7.6 |
| | | 15 | 15 | 100% | | |
| | | 14 | 14 | 100% | | |
| | 30JUN20 | 15 | 15 | 100% | 7.6 | 7.7 |
| | | 14 | 14 | 100% | | |
| | | 14 | 14 | 100% | | |
| | 06JUL20 | 15 | 15 | 100% | 7.5 | 7.6 |
| | | 15 | 15 | 100% | | |
| | | 15 | 14 | 93% | | |
| FCI | 17NOV20 | 15 | 15 | 100% | 7.0 | 7.4 |
| | | 15 | 15 | 100% | | |
| | | 15 | 15 | 100% | | |
| | 24NOV20 | 15 | 15 | 100% | 6.8 | 7.1 |
| | | 15 | 15 | 100% | | |
| | | 14 | 12 | 86% | | |
| | 01DEC20 | 10 | 10 | 100% | 6.7 | 7.5 |
| | | 10 | 10 | 100% | | |
| | | 10 | 10 | 100% | | |

**4 dpf Benzocaine**

| Facility | Pool | 4dpf | 5dpf | % Survival | pH before | pH after |
|---|---|---|---|---|---|---|
| **EPFL** | 27JUN20 | 10 | 0 | 0% | 7.6 | 7.7 |
| | | 14 | 0 | 0% | | |
| | | 13 | 0 | 0% | | |
| | 03JUL20 | 15 | 0 | 0% | 7.5 | 7.6 |
| | | 15 | 0 | 0% | | |
| | | 15 | 5 | 33% | | |
| | 26AUG20 | 15 | 0 | 0% | 7.6 | 7.3 |
| | | 15 | 0 | 0% | | |
| | | 13 | 0 | 0% | | |

# 2-Phenoxyethanol 3 mL/L

**EPFL:** 2-Phenoxyethanol, Sigma-Aldrich, 77699, USA

**FCI:** Aqua-Sed, VETARK, UK

### < 12 hpf 2-Phenoxyethanol

| Facility | Pool | <12hpf | <36hpf | % Survival | pH before | pH after |
|---|---|---|---|---|---|---|
| | | 15 | 1 | 7% | | |
| | 22JUN20 | 13 | 2 | 15% | 7.7 | 7.5 |
| | | 15 | 3 | 20% | | |
| | | 15 | 0 | 0% | | |
| **EPFL** | 01JUL20 | 13 | 0 | 0% | 7.6 | 7.4 |
| | | 15 | 0 | 0% | | |
| | | 15 | 0 | 0% | | |
| | 07JUL20 | 13 | 0 | 0% | 7.5 | 7.6 |
| | | 15 | 0 | 0% | | |

### 24 hpf 2-Phenoxyethanol

| Facility | Pool | 24hpf | 48hpf | % Survival | pH before | pH after |
|---|---|---|---|---|---|---|
| | | 15 | 15 | 100% | | |
| | 23JUN20 | 15 | 15 | 100% | 7.7 | 7.5 |
| | | 15 | 15 | 100% | | |
| | | 15 | 15 | 100% | | |
| **EPFL** | 30JUN20 | 14 | 14 | 100% | 7.6 | 7.7 |
| | | 16 | 16 | 100% | | |
| | | 15 | 15 | 100% | | |
| | 06JUL20 | 15 | 15 | 100% | 7.5 | 7.4 |
| | | 15 | 15 | 100% | | |
| | | 15 | 15 | 100% | | |
| | 17NOV20 | 15 | 15 | 100% | 7 | 7.4 |
| | | 15 | 15 | 100% | | |
| | | 15 | 15 | 100% | | |
| **FCI** | 24NOV20 | 15 | 15 | 100% | 6.8 | 7.5 |
| | | 10 | 10 | 100% | | |
| | | 10 | 10 | 100% | | |
| | 01DEC20 | 10 | 10 | 100% | 6.7 | 7.5 |
| | | 10 | 10 | 100% | | |

**4 dpf 2-Phenoxyethanol**

| Facility | Pool | 4dpf | 5dpf | % Survival | pH before | pH after |
|---|---|---|---|---|---|---|
| EPFL | 27JUN20 | 15 | 14 | 93% | 7.6 | 7.5 |
| | | 10 | 8 | 80% | | |
| | | 13 | 11 | 85% | | |
| | 03JUL20 | 15 | 14 | 93% | 7.5 | 7.6 |
| | | 15 | 15 | 100% | | |
| | | 15 | 15 | 100% | | |
| | 26AUG20 | 15 | 14 | 93% | 7.6 | 7.3 |
| | | 15 | 13 | 87% | | |
| | | 15 | 14 | 93% | | |

# Electrical Stunning

## 24 hpf Electrical stunning

| Facility | Pool | 24hpf | 48hpf | % Survival |
|---|---|---|---|---|
| **FCI** | 15DEC20 | 15 | 0 | 0% |
| | | 15 | 0 | 0% |
| | | 15 | 0 | 0% |
| | | 15 | 0 | 0% |
| | | 15 | 0 | 0% |

## 4 dpf Electrical stunning

| Facility | Pool | 4dpf | 5dpf | % Survival |
|---|---|---|---|---|
| **FCI** | 15DEC20 | 16 | 0 | 0% |
| | | 13 | 0 | 0% |
| | | 14 | 0 | 0% |
| | | 13 | 0 | 0% |
| | | 16 | 0 | 0% |
| | 08DEC20 | 15 | 0 | 0% |
| | | 15 | 0 | 0% |
| | | 15 | 0 | 0% |
| | | 15 | 0 | 0% |
| | | 15 | 0 | 0% |
| | | 15 | 0 | 0% |
| | | 15 | 0 | 0% |
| | | 15 | 0 | 0% |
| | | 15 | 0 | 0% |
| | | 22 | 0 | 0% |

# Hypothermic shock

## < 12 hpf Hypothermic shock

| Facility | Pool | <12hpf | <36hpf | % Survival |
|---|---|---|---|---|
| IP | 16NOV20 | 45 | 34 | 76% |
| | 23NOV20 | 45 | 40 | 89% |
| | 30NOV20 | 45 | 39 | 87% |
| UAB | 27OCT20 | 10 | 5 | 50% |
| | | 10 | 2 | 20% |
| | | 10 | 4 | 40% |

## 24 hpf Hypothermic shock

| Facility | Pool | 24hpf | 48hpf | % Survival |
|---|---|---|---|---|
| IP | 16NOV20 | 45 | 39 | 87% |
| | 23NOV20 | 45 | 40 | 89% |
| | 30NOV20 | 45 | 26 | 58% |
| UAB | 27OCT20 | 10 | 10 | 100% |
| | | 10 | 10 | 100% |
| | | 10 | 9 | 90% |

## 4 dpf Hypothermic shock

| Facility | Pool | 4dpf | 5dpf | % Survival |
|---|---|---|---|---|
| IP | 26NOV20 | 44 | 44 | 100% |
| | 23NOV20 | 45 | 45 | 100% |
| | 30NOV20 | 45 | 45 | 100% |
| UAB | 27OCT20 | 10 | 10 | 100% |
| | | 10 | 10 | 100% |
| | | 10 | 10 | 100% |
